# Atomic optical stimulated amplifier with optical filtering of ultra-narrow bandwidth

**DOI:** 10.1038/s41598-018-24895-x

**Published:** 2018-04-26

**Authors:** Duo Pan, Tiantian Shi, Bin Luo, Jingbiao Chen, Hong Guo

**Affiliations:** 1 State Key Laboratory of Advanced Optical Communication Systems and Networks, School of Electronics Engineering and Computer Science, and Center for Quantum Information Technology, Peking University, Beijing, 100871 China; 2 State Key Laboratory of Information Photonics and Optical Communications, Beijing University of Posts and Telecommunications, Beijing, 100876 China

## Abstract

Taking advantages of ultra-narrow bandwidth and high noise rejection performance of the Faraday anomalous dispersion optical filter (FADOF), simultaneously with the coherent amplification of atomic stimulated emission, we propose a stimulated amplified Faraday anomalous dispersion optical filter (SAFADOF) at cesium 1470 nm. The SAFADOF is able to significantly amplify very weak laser signals and reject noise in order to obtain clean signals in strong background. We show that for a weak signal of 50 pW, the gain factor can be larger than 25000 (44 dB) within a bandwidth as narrow as 13 MHz. Having the ability to amplify weak signals with low background contribution, the SAFADOF finds outstanding potential applications in weak signal detections.

## Introduction

The Faraday anomalous dispersion optical filter (FADOF)^1,2^ has advantages of ultra-narrow bandwidth^3^, high transmittance, and high noise rejection^4,5^, which makes it an excellent frequency selection component widely used in optical signal processing^6–9^ and more generally, in weak optical communication, such as free-space optical communication^10^ and underwater optical communication^11^. Typically, in free-space quantum key distribution (QKD) systems^12,13^ and lidar remote sensing systems^14–17^, narrow-bandwidth FADOFs are usually used to suppress out-of-band noise, thus reducing the error rate and enable observations in strong background. In such systems, the ability to extract weak signal from strong background noise relies on the narrow bandwidth of the filters, and meanwhile, the total transmission efficiency is proportional to the FADOFs’ transmittance. Therefore, to enable applications in longer communication distance and higher accuracy, conventional FADOFs have been developing towards the trend of higher transmittance and narrower bandwidth.

Up to now, the FADOFs have been realized on different atomic transitions, mostly with transmittance between 40% and 100%, and equivalent noise bandwith (ENBW) around 1 GHz, such as Na 589 nm (90%, 5 GHz)^18^, Rb 780 nm (83%, 2.6 GHz)^19^, Rb 795 nm (70%, 1.2 GHz)^20^, Cs 459 nm (98%, 1.2 GHz)^21^, Cs 852 nm (88%, 0.56 GHz)^22^, Cs 894 nm (77%, 0.96 GHz)^23^, Sr 461 nm (63%, 1.19 GHz)^24^, etc^25–28^. An ultra-narrow optical filter based on Faraday effect has been demonstrated in 2012^29^, of which the bandwidth is 6.2 MHz. However, the transmittance of this filter is only 9.7%, which finally limited its application. To break the restriction of transmittance, an atomic filter with Raman light amplification has been studied^30–32^, in which a Raman light amplifier and a FADOF are used in tandem with independent Rb cells. This filter enhanced the transmittance to 85-fold compared to the case operating only with the FADOF, which expands the range of potential applications. However, for ultra weak signal detection, the amplification is still unable to meet the requirement. Also, the ability to suppress the background noise is determined by the FADOF bandwidth of 0.6 GHz, which is limited by the atomic Doppler broadening.

Here, we demonstrate a stimulated amplified Faraday anomalous dispersion optical filter (SAFADOF) at 1470 nm, which realizes the high noise rejection performance of the FADOF and the coherent amplification^33^ of atomic stimulated emission simultaneously in a single Cs atomic cell. By this means an atomic filter based on population inversion is realized, and the stimulated emission process provides quite effective amplification as well as an ultra-narrow bandwidth. Experimentally, we measure a gain factor larger than 25000 (44 dB) with a probing light power of 50 pW. An ultra-narrow full width at half maximum (FWHM) of 13 MHz is achieved, and the out-band noise is totally rejected with a noise rejection ratio of 1 × 10^5^. Being much more efficient in extracting weak signals from strong background compared with any existing atomic filters, the SAFADOF provides quite promising applications in weak signal detection in optical communication.

## Methods

### Experimental apparatus

**Figure 1:**
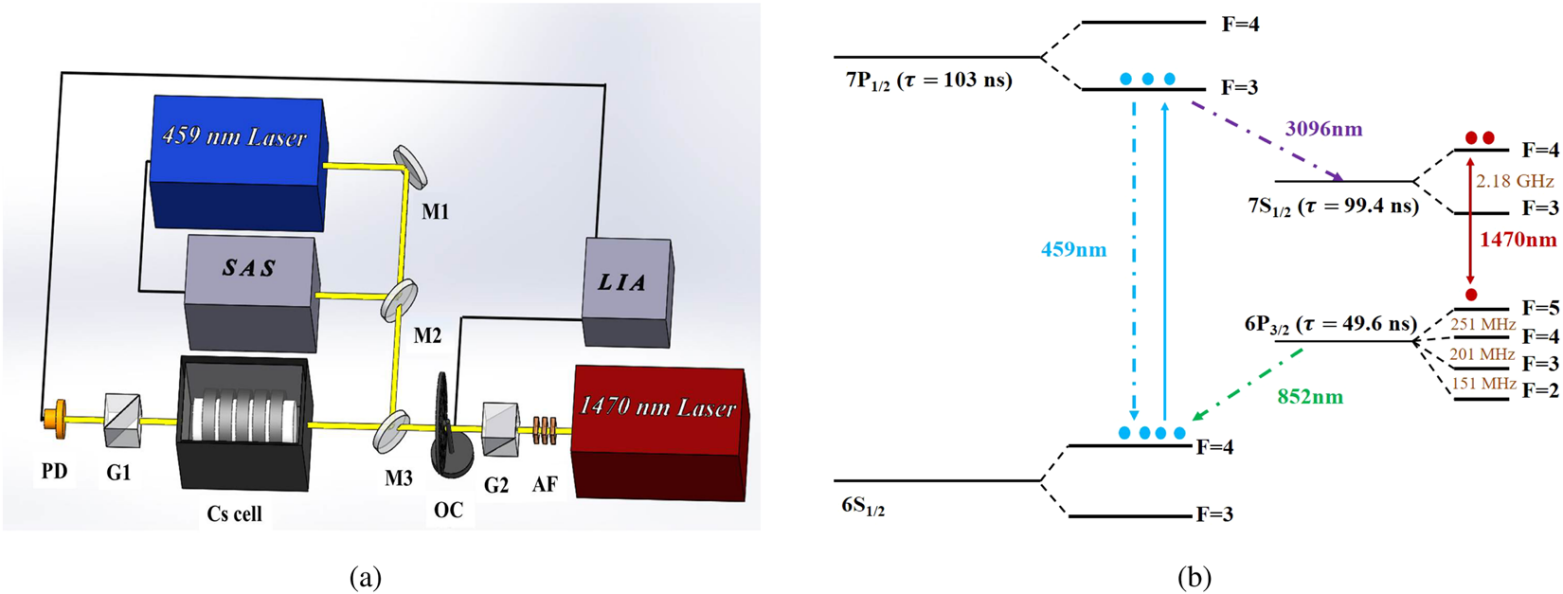
(**a**) Experimental setup of the SAFADOF. SAS: saturated absorption spectroscopy. LIA: lock-in amplifier. OC: optical chopper. AF: Attenuation filters. M1: 459 nm high-reflecting mirror. M2: 459 nm partially-reflecting mirror. M3: 459 nm high-reflecting and 1470 nm anti-reflecting mirror. G1 and G2: a pair of Glan-Taylor prisms whose polarization directions are orthogonal. PD: Photo diode. (**b**) The related energy levels of Cs atom.

The experimental setup and relevant energy level structures are shown in Fig. 1. A 459 nm laser stabilized to the Cs 6*S*_1/2_(*F* = 4) − 7*P*_1/2_(*F* = 3) transition by the saturated absorption spectroscopy (SAS) pumps the Cs atoms inside a 10 cm-long quartz cell. The pumping laser corresponds to a weak transition whereafter the spontaneous decay of the excited state will occur via multiple intermediate states, and the analogous energy structure has been studied in various systems^34–37^. After pumping, the Cs atoms are population inverted between 7*S*_1/2_(*F* = 4) and 6*P*_3/2_(*F* = 5) states^38^. Hence with the function of the 1470 nm probing laser (coincide with the pumping laser), stimulated emission between the two states is generated, and thus the probing laser is significantly amplified. The Cs cell is placed between a pair of orthogonal Glan-Taylor prisms G1 and G2, of which the extinction ratio is 1 × 10^5^ and drops to 6 × 10^4^ with the vapor cell and the dichroic mirror between them. This also determines the out-of-band noise rejection ratio of the SAFADOF. The ring magnets outside the cell produce an axial magnetic field of about 8 Gauss, where we experimentally get the largest gain. An optical chopper together with a lock-in amplifier are used to eliminate the influences of the fluorescence generated by static superradiance^39,40^.

### Theory calculation of the gain factor

Considering SA operation, the stimulated emission can enhance the input signal with a factor of *G*_SA_. Combined with the function of two crossed Glan-Taylor prisms, the SAFADOF gain is given by:

1 $$G={G}_{SA}\times {\sin }^{2}\,\phi ,$$

where the rotation angle is given by

2 $$\begin{array}{rcl}\phi =\frac{\pi l}{\lambda }({n}_{+}-{n}_{-}) & = & \frac{\pi l}{2\lambda }\,{\rm{Re}}({{\chi }}_{+}-{{\chi }}_{-})\\  & = & \frac{3N{\rm{\Gamma }}{\lambda }^{2}l}{8\pi }\frac{{g}_{F}{\mu }_{B}B/\hslash }{{({g}_{F}{\mu }_{B}B/\hslash )}^{2}+{({\rm{\Gamma }}\mathrm{/2})}^{2}}.\end{array}$$

Here the relaxation rate Γ = 55 MHz, considering the natural broadening as well as the Doppler broadening caused by the saturated pumping^38^. The calculation method is described in refs^38,41^, and the detailed meaning of the parameters in Eq. (2) is given in ref.^42^. For *φ* ≤ *π*/2, sin^2^ *φ* has the maximum value when *g*_*F*_*μ*_*B*_*B*/*ħ* = Γ/2, thus we have *B* ≈ 7.8 *G*. Experimentally we get the maximum gain at *B* ≈ 8 *G*, and the transmitted ratio is closed to 100% at 135 °C, meaning that we get almost the same gain factor with or without the two crossed Glan-Taylor prims. For simplicity, we keep the magnetic field to be optimal and assume:

$$G={G}_{SA}$$

when calculating the gain factor at 135 °C.

For the interaction of a two-level atomic system with a radiation field, the transition probability is given by *W*(*t*) = |*c*(*t*)|^2^, with $c(t)=-\,i\frac{{\rm{\Omega }}}{\sqrt{{{\rm{\Omega }}}^{2}+{\rm{\Delta }}{\omega }^{2}}}\,\sin \,(\frac{\sqrt{{{\rm{\Omega }}}^{2}+{\rm{\Delta }}{\omega }^{2}}}{2}t)\,\exp \,[-i\frac{{\rm{\Delta }}\omega }{2}t]$^43^, where Ω and Δ*ω* represent the Rabi frequency and the frequency detuning respectively. Thus for a radiation field on resonance, the transition probability is expressed as

3 $$W(t)={\sin }^{2}\,\frac{{\rm{\Omega }}t}{2}.$$

For the atoms with average lifetime *τ*, the distribution function of their interaction time with the radiation field is represented in the form $f(t)=\tfrac{1}{\tau }{e}^{-t/\tau }$. Then Eq. (3) transforms into

4 $$\langle W\rangle ={\int }_{0}^{\infty }\,f(t)W(t)dt=\frac{1}{2}\frac{{{\rm{\Omega }}}^{2}}{{{\rm{\Omega }}}^{2}+{{\rm{\Gamma }}}^{2}},$$

again Γ is the relaxation rate considering the spontaneous emission and the Doppler broadening^38^. To match our experimental conditions, considering the length of the Cs cell and the probing laser with waist *w*_0_, the variation of signal power d*P* during a length of d*L* is given by

5 $${\rm{d}}P=\frac{1}{2}\eta {\rm{\Delta }}\rho \pi {w}_{0}^{2}h\nu \times \frac{{{\rm{\Omega }}}^{2}}{{{\rm{\Omega }}}^{2}+{{\rm{\Gamma }}}^{2}}{\rm{d}}L,$$

where *η* = 1/*τ*_*cyc*_ represents the pumping rate, with *τ*_*cyc*_ being the atomic cycling time. Analogous to ref.^44^, for Cs atoms we have

$${\tau }_{cyc}=\frac{1}{{\rm{\Omega }}}+\frac{1}{{{\rm{\Gamma }}}_{23}+{{\rm{\Gamma }}}_{24}}+\frac{1}{{{\rm{\Gamma }}}_{35}+{{\rm{\Gamma }}}_{36}}+\frac{1}{{{\rm{\Gamma }}}_{51}},$$

with Γ_23_, Γ_24_, Γ_35_, Γ_36_, Γ_51_ corresponding to the relaxation rates of transitions 7*P*_1/2_ − 7*S*_1/2_, 7*P*_1/2_ − 5*D*_3/2_, 7*S*_1/2_ − 6*P*_3/2_, 7*S*_1/2_ − 6*P*_1/2_, and 6*P*_1/2_ − 6*S*_1/2_, respectively. Some of the energy levels are not displayed in Fig. 1(b), referring to ref.^38^. In our experiment *η* is calculated to be 3.6 × 10^6^/*s*. The effective atomic density Δ*ρ* is estimated according to the experimental parameters. Taking into account the atomic distribution in thermal equilibrium being 1.0 × 10^20^/*m*^3^ at 135 °C^45^, and the population inversion ratio being about 0.03^38^, the atomic density difference in the 7*S*_1/2_(*F* = 4) and 6*P*_3/2_(*F* = 5) states is 3.0 × 10^18^/*m*^3^. Then with the Doppler broadening, only the atoms having Doppler-shifted frequency detuning within the linewidth of 1470 nm probing laser (about 300 kHz) are efficient to amplify the probing laser signal, resulting in an effective atomic density of 3.4 × 10^15^/*m*^3^.

Hence by integrating the expression through the interaction region L, we obtain the equation with the help of ${{\rm{\Omega }}}^{2}=\tfrac{{{\rm{\Gamma }}}^{2}}{2}\times \tfrac{{\rm{I}}}{{{\rm{I}}}_{s}}$^46^ as:

6 $$P-{P}_{0}+2\pi {w}_{0}^{2}{I}_{s}\times \,\mathrm{ln}\,\frac{P}{{P}_{0}}=\frac{1}{2}\eta {\rm{\Delta }}\rho \pi {w}_{0}^{2}h\nu L,$$

where ${I}_{s}=\tfrac{h\pi c{\rm{\Gamma }}}{3{\lambda }^{3}}$^46^, is the saturation intensity, and *P*_0_ is the input probing light power.

Considering *G* = *P*/*P*_0_, we have

7 $${P}_{0}\times (G-\mathrm{1)}+2\pi {w}_{0}^{2}{I}_{s}\times \,\mathrm{ln}\,G=\frac{1}{2}\eta {\rm{\Delta }}\rho \pi {w}_{0}^{2}h\nu L\mathrm{.}$$

By Eqs (6) and (7) we obtain the theoretical transmitted power as well as the gain factor depending on the probe power at 135 °C as depicted in Fig. 3(d), which will be analyzed in the following.

## Results

### Superradiance background

Due to the collective behavior of static superradiance^39,40^, population reversed atom ensemble will radiate spontaneously from the 7*S*_1/2_(*F* = 4) state to the 6*P*_3/2_(*F* = 5) state, which is much faster and stronger than that of individual atoms, and exhibit well defined direction. In our system the 1470 nm static superradiance has been observed experimentally^39^.

**Figure 2:**
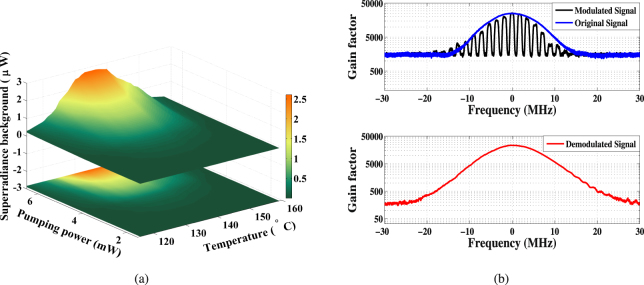
(**a**) Detected superradiance background for various temperatures and pumping powers. (**b**) The transmission signal before and after modulation (up) and the demodulated signal (down).The results are obtained by scanning the laser frequency.

The static superradiance light, of which the amplitude varies with the pumping power and temperature, cannot be optically filtered and will contribute to the background noise, as shown in Fig. 2(a). Such influence is eliminated by a synchronous modulation method, where the probing light is pre-modulated by an optical chopper, with a modulation frequency of 1.5 kHz. Then the detected transmission light is demodulated by a lock-in amplifier synchronized to the chopper. So that the transmission signal derived from the probing laser is well separated from the static superradiance and independently detected. Figure 2(b) illustrates the gain spectrum before and after modulation, as well as the demodulated signal in which the background is effectively suppressed. This method is proposed to improve the SNR of the SAFADOF, and is also applicative in other systems such as lamp-based atomic filters^24,47^, where the fluorescence has non-negligible influence.

### Gain factor

In the context of weak optical communication, we are interested in obtaining long communication distance and high accuracy, which requires a high transmittance of the filter to reduce the loss, or possibly, a high gain factor. Compared to the above-mentioned Raman amplified atomic filter, where the Raman gain is transformed from the coupling laser without population inversion, the SAFADOF provides much more effective amplification.

**Figure 3:**
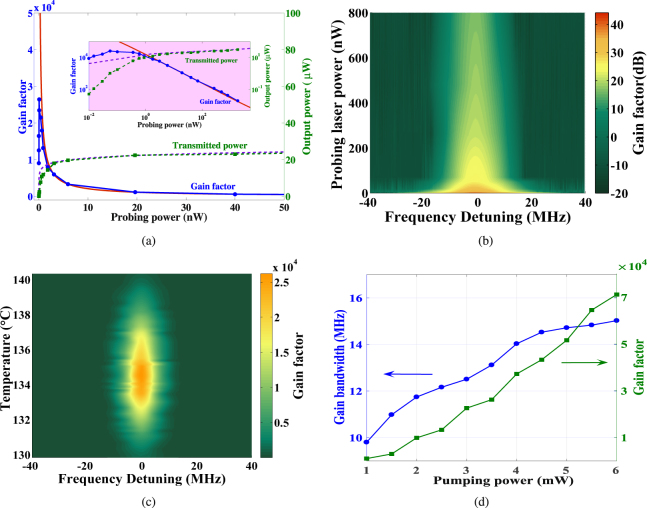
(**a**) Calculated (red, solid) and measured (blue, solid) gain factor, as well as calculated (purple, dashed) and measured (green, dashed) transmission power of the SAFADOF for various probing powers. (**b**) Density plot of the gain spectrum (in dB) for various probing powers at 135 ºC. (**c**) Gain spectrum for different temperature at the probing power of 50 pW. (**d**) Experimental gain bandwidth together with the measured gain factor as a function of pumping power.

Figure 3(a) displays the calculated transmitted power at resonance (purple, dashed) and gain factor (red, solid) as a function of the probing power at 135 °C with 3.5 mW 459 nm pumping power. We see that the transmitted power quickly tends to a saturation value due to the limited output capability of the atoms, thereafter the gain factor decreases in an approximate inverse proportional relationship to the probing power. Experimentally the measured transmitted power (green, dashed) and gain factor (blue, solid) are also depicted. For probing powers relatively large, the measured results agree well with the calculation, while for ultra weak probing powers the measured gain factor undergoes a sharp decline. It may because that in this case the superradiance effect plays a larger role and the lock-in technique is not sufficient to separate the two effects. The largest gain factor of more than 25000 (44 dB) is obtained at 50 pW. For various probing powers and temperatures, the gain spectrums are density plotted in Fig. 3(b,c) respectively. While the gain factor decreases for lower temperature due to the reduction of the Cs atomic density in the cell, for higher temperature the increased collisions between atoms decrease the coherence time of the 7*S*_1/2_ state, thus decreasing the gain factor. Such characteristics have also been reported in hydrogen maser^48^, Rb and Cs atomic systems^38,39,49^.

### Gain bandwidth

The gain bandwidth is of interest in particular regarding suppression requirement of the background noise. In the SAFADOF, the gain bandwidth is approximate to the natural linewidth of the atomic transition, for the zero-velocity selection of the atoms by Doppler-free stabilized pumping laser. However, as the power of the pumping laser increases, the saturation effect results in a velocity distribution of Cs atoms pumped to the 7*P*_1/2_ state. These atoms decays to the 7*S*_1/2_ state and participate in the stimulation emission, and finally broadens the gain bandwidth of the SAFADOF through the Doppler effect. Figure 3(d) shows the dependency on pumping power of the gain factor and the gain bandwidth. We see that with the pumping power increasing, a larger gain factor is obtained. Meanwhile the gain bandwidth is broadened, which indicates that there is some optimal pumping power depending on how large the gain factor is required. The preferred pumping power will depend on the particular application.

## Conclusion

In summary, we have experimentally investigated a SAFADOF at 1470 nm based on population inversion. The SAFADOF provides a gain factor larger than 25000 (44 dB) and an ultra-narrow bandwidth of 13 MHz, and it opens the possibility of applications in weak optical communication.

To eliminate the fluorescence background caused by superradiance of the Cs atoms, we propose a synchronous modulation method, which experimentally suppressed the background, and the method can be further expanded to other lamp-based atomic filters^24,47^. We also studied the gain factor and gain bandwidth characteristics of the SAFADOF under different probing laser powers, pumping laser powers, and temperatures. The gain factor has an approximate inverse proportional relationship with the probing power, and increases with the pumping power, while the gain bandwidth mainly increases with the pumping power. Hence a trade-off between large gain factor and narrow bandwidth must be made when determining the pumping power in practice.
